# Intention‐to‐Leave Among New Zealand Podiatrists: A Cross‐Sectional Survey of Frequency and Associated Factors

**DOI:** 10.1002/jfa2.70236

**Published:** 2026-10-21

**Authors:** Mia Clarke, Mike Frecklington, Irene Zeng, Matthew R. Carroll, Richard J. Siegert, Sarah Stewart

**Affiliations:** ^1^ Department of Podiatry School of Allied Health Auckland University of Technology Auckland New Zealand; ^2^ Biostatistics Department of Clinical Sciences Management Auckland University of Technology Auckland New Zealand; ^3^ Department of Psychology and Neuroscience School of Science Auckland University of Technology Auckland New Zealand

**Keywords:** attrition, health workforce, intention to leave, New Zealand, podiatry, retention, turnover

## Abstract

**Background:**

Workforce retention poses ongoing challenges for podiatric care provision in Aotearoa New Zealand (NZ); however, intention‐to‐leave (ITL) from the workforce and its associated factors have not been explored. This study aimed to determine the frequency of ITL among NZ podiatrists and identify factors associated with leaving intentions.

**Methods:**

A cross‐sectional online survey of NZ‐registered practising podiatrists was conducted. Demographics, employment, workplace and attitudinal measures were collected as independent variables. ITL was assessed using (1) a self‐reported single‐item Likert scale and (2) total scores on the validated six‐item Turnover Intention Scale (TIS‐6), with higher scores indicating greater turnover intention. Associations between both ITL outcomes and independent variables were examined using theory‐informed hierarchical logistic and linear regression models, as appropriate.

**Results:**

A total of 143 podiatrist responses were analysed. Over half (50.7%) reported being somewhat or highly likely to leave the profession, with 23.8% indicating plans to exit within 2 years. The mean TIS‐6 total score was 15.41/30 (SD 5.16). In fully adjusted models, workplace and psychosocial factors demonstrated the most consistent associations with ITL. Lower organisational support (OR = 0.60, *p* = 0.049), lower role satisfaction (OR 0.64, *p* = 0.041; *B* = −2.08, *p* ≤ 0.001), lower perceived professional respect (*B* = −1.14, *p* < 0.05) and higher stress levels (OR 0.57, *p* = 0.023) were associated with greater ITL.

**Conclusions:**

ITL among NZ podiatrists is prevalent, with over half of this sample reporting some ITL and almost one quarter considering a near‐term exit. Given the relatively small NZ podiatry workforce, these findings raise important concerns regarding future workforce sustainability and service‐provision capacity. Addressing modifiable factors such as role satisfaction, organisational support, professional value and workplace stress may represent important targets for retention initiatives.

## 1 Introduction

Podiatry is an essential allied health service that contributes to the management of foot and lower limb pathology associated with long‐term conditions, injury, and age‐related changes [1]. These services are central to maintaining mobility, preventing complications, and reducing the downstream burden on the health system [2, 3, 4]. Despite this, the podiatry workforce in Aotearoa New Zealand (NZ) remains small and unevenly distributed, with only 460 podiatrists holding a NZ Annual Practising Certificate (APC) in 2025 [5]. Workforce shortages contribute to service gaps and inequities in access to care, disproportionately affecting rural, Māori, and other priority populations [6, 7, 8].

Intention‐to‐leave (ITL), defined as a worker's self‐reported likelihood of leaving their organisation or profession, is a well‐established predictor of workforce attrition [9, 10]. In small, capacity‐constrained professions such as podiatry, elevated ITL poses a significant risk to service continuity and workforce sustainability.

An Australian survey found that approximately one‐fifth of podiatrists intended to leave the profession within 5 years, with intentions associated with unfavourable working conditions, burnout, and lower professional satisfaction [11]. Although the prevalence of ITL among NZ podiatrists remains unknown, several factors known to be associated with workforce leaving intentions in other populations have been identified. A 2023 national survey of NZ podiatrists reported high levels of burnout risk and occupational stress associated with long working hours and collegial isolation [12], while qualitative research among rural NZ podiatrists identified professional isolation and excessive workloads as barriers to recruitment and workforce retention [6]. These findings suggest that several important drivers of ITL exist in NZ, though their relative contributions remain unclear.

Workforce growth within NZ podiatry remains minimal. Between 2020 and 2025, the number of podiatrists holding a current APC increased by only three practitioners (< 1% workforce growth) [5]. This minuscule increase has been substantially outpaced by population growth and projected rises in chronic disease prevalence [13, 14], suggesting workforce leakage, whereby graduate inflow and immigration have not translated into sustained workforce expansion [8]. Additionally, international comparisons indicate that NZ would require more than double its current podiatry workforce to achieve practitioner‐to‐population ratios comparable to those of the United Kingdom and Australia [8].

While increasing podiatry student enrolments is often proposed to address workforce shortages, retaining the existing workforce and its cumulative clinical experience is equally important. However, no national quantitative study has examined the extent of ITL among NZ podiatrists or identified factors associated with these intentions. Addressing this knowledge gap is essential to inform targeted retention strategies and support workforce growth and sustainability. Therefore, the objectives of this study were to: (i) determine the frequency of ITL among podiatrists in NZ; and (ii) identify factors associated with ITL among podiatrists in NZ.

## 2 Methods

### 2.1 Design

This cross‐sectional study used data from an anonymous online survey of podiatrists practising in NZ between April and October 2025. Reporting followed the Checklist for Reporting Results of Internet E‐Surveys (CHERRIES) [15]. Ethical approval was obtained from the Auckland University of Technology Ethics Committee (Reference 25/91).

### 2.2 Participants and Setting

The survey was open to all practising podiatrists in NZ who held a current APC and were registered with the Podiatry Board of NZ (PBNZ) during the study period. At the time of data collection, approximately 460 podiatrists met these criteria [5]. A voluntary response sampling approach was used. Recruitment was conducted through advertisements in the Podiatry NZ e‐newsletter and relevant social media platforms, as well as through promotion at regional podiatry conferences.

### 2.3 Data Collection and Procedures

Data were collected using an online Qualtrics survey (Qualtrics, Provo, UT, USA). The survey link directed participants to an information page outlining the study purpose, eligibility criteria, and consent process. Forced‐response settings and skip logic were applied to minimise missing data; however, participants could withdraw at any time prior to submission. Upon completion, participants could enter a prize draw for a gift voucher.

The survey captured demographic, employment, workplace, attitudinal, and ITL data. Survey content was informed by established correlates of ITL and factors relevant to the NZ podiatry context [16, 17, 18]. Items were primarily categorical multiple‐choice questions, with optional free‐text responses for selected items. The survey was pilot‐tested with five practising NZ podiatrists, resulting in minor revisions for improved clarity and flow. The mean completion time was approximately 10 minutes.

The final survey comprised four sections (Supporting Information S1): (1) demographic characteristics, (2) employment characteristics, (3) workplace and attitudinal measures, and (4) ITL measures. Demographic variables included gender, age, ethnicity, marital status, dependants, household income contribution, geographical location, and years in practice. Employment variables included practice setting, role, qualifications, further study intentions, patient caseload, and work hours. Workplace and attitudinal variables assessed satisfaction, psychological strain, and perceived professional respect and support. ITL was assessed using two related but conceptually distinct measures. Self‐reported ITL was measured using a single‐item five‐point Likert scale ranging from ‘highly unlikely’ to ‘highly likely’. ITL was also assessed using the Turnover Intention Scale‐6 (TIS‐6), a validated six‐item measure of cognitive turnover intention [19]. The TIS‐6 demonstrates good internal consistency (Cronbach's *α* = 0.80) and construct validity across occupational settings [19, 20, 21]. Internal consistency was similarly good in the present sample (Cronbach's *α* = 0.83). Unlike the single‐item measure, the TIS‐6 captures broader dimensions of turnover cognition, including thoughts of leaving, consideration of alternative employment, and cognitive orientation toward departure. The inclusion of both outcomes enabled examination of anticipated profession exit alongside broader dimensions of turnover intention.

Each TIS‐6 item is rated on a five‐point Likert scale, with total scores ranging from 6 to 30; higher scores indicate greater turnover intention [19]. The continuous total score was used as the primary TIS‐6 outcome, as it preserves variability and allows assessment across the full spectrum of leaving intentions. TIS‐6 scores have also been dichotomised in previous work using validated cut‐points representing low (< 18) and high (≥ 18) ITL [20, 22].

Participants reporting neutral or likely leaving intentions on the single‐item scale completed additional items regarding their anticipated timeframe for exit and perceived reasons for leaving. Reasons were assessed using a multiple‐response format and included workplace, professional, and personal factors.

## 3 Statistical Analyses

### 3.1 Objective i: Frequency of ITL

Descriptive statistics summarised ITL as a categorical and a continuous outcome. Frequency was reported as *n* (%) using the ordinal single‐item Likert measure and the continuous TIS‐6 total score using mean (SD) and median (IQR). For descriptive purposes, TIS‐6 scores were also summarised using previously validated low (< 18) and high (≥ 18) ITL categories and reported as *n* (%).

### 3.2 Objective ii: Factors Associated With ITL

In regression analyses, ITL was measured using the ordinal single‐item Likert measure and continuous TIS‐6 score, which were treated as dependent variables. Participant demographics, employment, workplace, and attitudinal characteristics were considered independent variables. Free‐text responses were inductively coded and aligned with existing categories. Independent variables were coded according to an a priori analytical framework: ordinal variables were entered as numeric where appropriate, nominal variables were treated as categorical or dummy‐coded, and sparse categories were collapsed where necessary to ensure model stability. Variables representing proximal indicators of ITL (e.g., intended timeframe to leave, reasons for leaving, willingness to recommend podiatry) were excluded from multivariable regression modelling because of conceptual overlap with the outcome measures, but are reported descriptively.

Theory‐informed hierarchical (sequential) regression models were specified a priori [23], consistent with conceptual turnover models in which intentions develop from distal demographic characteristics through employment conditions to proximal attitudinal and psychological responses [24, 25, 26, 27, 28]. Independent variables were entered sequentially in four blocks (demographic, employment, workplace, and attitudinal) to evaluate the combined contribution of conceptually related factors while preserving theoretical structure [29, 30]. Model specification prioritised theoretical relevance, interpretability, and minimisation of collinearity while balancing model complexity relative to sample size. Given the exploratory and observational nature of the analyses, hierarchical models were intended to identify broader patterns of association rather than to precisely predict individual subgroup effects.

To strengthen the robustness of findings, complementary modelling approaches were employed, consistent with triangulation frameworks that advocate comparing theory‐driven and data‐driven analytical strategies [31]. In addition to the primary theory‐informed hierarchical models, forward stepwise models based on the Akaike Information Criterion (AIC) were fitted to assess whether key findings remained broadly consistent under an alternative data‐driven model‐building approach. Post‐stratification weights based on the age and sex distribution of the NZ podiatry workforce were applied to the primary hierarchical models to assess the potential influence of sampling and non‐response bias [5, 32, 33]. Consistency of findings across these approaches was interpreted as strengthening confidence in the robustness of the findings.

Data were screened prior to inferential analysis and model assumptions were assessed before interpretation. Complete‐case analysis was used for multivariable hierarchical modelling to maintain consistency across sequential blocks. Consequently, final sample sizes varied across models based on the availability of complete data for included independent variables. Diagnostic procedures included assessing multicollinearity, model fit, residual behaviour, influence statistics, and proportional‐odd assumptions (where applicable). Assumption checks indicated no material violations across fitted models, and findings remained substantively consistent across alternative model specifications. Further details are provided in Supporting Information S2.

Statistical significance was set at *p* < 0.05 unless otherwise specified. Given the exploratory nature of the analysis, multiplicity adjustments were not applied at the multivariable stage; all estimates, tests, and *p‐*values are reported. All descriptive, bivariate, and post‐stratification analyses were conducted in IBM SPSS Statistics (v.31), while regression modelling was performed in RStudio (v.2025.05.0‐496).

#### 3.2.1 Factors Associated With ITL (Single‐Item Likert Measure)

Univariable proportional‐odds ordinal logistic regression models were initially fitted to screen independent variables for multivariable modelling. These analyses were used to inform multivariable model building rather than for inference regarding individual associations. To account for multiple comparisons at this stage, likelihood‐ratio (LR) *p*‐values were adjusted using the Benjamini–Hochberg false discovery rate procedure [34]. Independent variables with unadjusted LR *p*‐values of *p* < 0.20 were considered for multivariable modelling [35].

Multivariable analyses were conducted using hierarchical proportional‐odds ordinal logistic regression. Results are presented as odds ratios (ORs) with 95% confidence intervals (CIs), representing the odds of greater ITL. As a complementary modelling approach, forward stepwise ordinal logistic regression using AIC selection was also performed. Relative importance metrics were calculated for the final stepwise model to aid interpretation.

#### 3.2.2 Factors Associated With TIS‐6 Scores

Bivariate analyses examined associations between TIS‐6 scores and independent variables using parametric or nonparametric methods as appropriate. Similarly, these analyses were used to inform multivariable model building rather than for inference regarding individual associations. Independent variables with *p* < 0.10 were considered for multivariable modelling [36]. Multiple linear regression models were fitted using the same hierarchical block structure described above. Regression coefficients (*β*) represent adjusted mean change in TIS‐6 score, with positive values indicating greater ITL. As a complementary modelling approach, forward stepwise linear regression using AIC selection was also performed.

## 4 Results

### 4.1 Sample Characteristics

A total of 164 podiatrists responded to the survey. After excluding incomplete and ineligible responses, 143 participants were included in the final analysis (completion rate 87.2%), representing approximately 35% of the NZ podiatry workforce [5].

Demographic characteristics are summarised in Table 1. Most participants were female, NZ European, married, and aged 26–40 years. Gender and ethnicity distributions were representative of the national workforce [5]. Over half of the participants had dependants, and most contributed the majority of their household income. Participants were primarily located in major metropolitan regions (Auckland, Wellington, and Canterbury), and most had more than 11 years of practice experience. Employment characteristics (Table 2) indicated that over 90% worked in private practice, with fewer than one‐fifth also employed in the public sector. Clinic owners comprised the largest employment group. More than one‐third reported working in isolation and over 40 hours per week.

**TABLE 1:** Participant characteristics (*n* = 143).

Variable	Summary statistics, *n*(%)
Gender	
Male	37 (25.9%)
Female	105 (73.4%)
Other gender	NR^a^
Age	
20–25 years	13 (9.1%)
26–30 years	23 (16.1%)
31–35 years	19 (13.3%)
36–40 years	24 (16.8%)
41–45 years	10 (7.0%)
46–50 years	13 (9.1%)
51–55 years	14 (9.8%)
56–60 years	11 (7.7%)
61–65 years	8 (5.6%)
66 years or older	8 (5.6%)
Ethnicity^b^	
NZ European	104 (72.7%)
Māori	10 (7.0%)
Asian	14 (9.8%)
Pacific	4 (2.8%)
Middle Eastern, Latin American, African	2 (1.4%)
Other	17 (11.9%)
Marital status	
Single	21 (14.7%)
In a relationship	17 (11.9%)
De facto partnership	14 (9.8%)
Married	82 (57.3%)
Divorced/separated	5 (3.5%)
Prefer not to say	4 (2.8%)
Number of children/dependants	
0	68 (47.6%)
1	12 (8.4%)
2–3	60 (42.0%)
4 or more	3 (2.1)
Household income contribution^c^ ^,^ ^d^	
Less than 25%	10 (7.0%)
25%–49%	30 (21.0%)
50%–74%	48 (33.6%)
75%–100%	20 (14.0%)
Region of practice	
Northland	7 (4.9%)
Auckland	55 (38.5%)
Waikato	9 (6.3%)
Bay of Plenty; Gisborne	10 (7.0%)
Hawkes Bay	7 (4.9%)
Taranaki; Manawatu‐Wanganui	6 (4.2%)
Wellington	12 (8.4%)
Canterbury; Marlborough	29 (20.3%)
Otago	8 (5.6%)
Practice years	
Less than 2 years	10 (7.0%)
2–5 years	31 (21.7%)
6–10 years	16 (11.2%)
11–15 years	23 (16.1%)
16–20 years	11 (7.7%)
More than 20 years	52 (36.4%)

Abbreviation: NR = Not reported.

^a^ Categories with *n* = 1 were suppressed to protect participant confidentiality in accordance with the Data and Statistics Act 2022.

^b^ Multiple choice, total may exceed sample size.

^c^ Answered only by participants with dependants or partners.

^d^ Percentages are presented relative to the total sample (*n* = 143) to provide a consistent denominator across all variables.

**TABLE 2:** Employment characteristics (*n* = 143).

Variable	Summary statistics, *n* (%)
Sector^a^	
Private practice	133 (93.0%)
Public	25 (17.5%)
Research	2 (1.4%)
Education	5 (3.5%)
Other	2 (1.4%)
Employment position^a^	
Owner/co‐owner	67 (46.9%)
Permanent employee	60 (42.0%)
Fixed‐term/casual	7 (4.9%)
Independent contractor	24 (16.8%)
Other	4 (2.8%)
Future ownership intent^b^	
Definitely not	16 (11.2%)
Probably not	21 (14.7%)
Might or might not	17 (11.9%)
Probably yes	12 (8.4%)
Definitely yes	10 (7.0%)
Secondary employment outside of podiatry	
Yes	16 (11.1%)
No	128 (88.9%)
Education	
Diploma	16 (11.2%)
Bachelor's	70 (49.0%)
Postgraduate diploma/certificate/honours	49 (34.3%)
Master's; doctoral	8 (5.6%)
Currently enrolled in tertiary education	
Yes	14 (9.8%)
No	129 (90.2%)
Future enrolment intent^c^	
Yes	32 (22.4%)
No	55 (38.5%)
Unsure	42 (29.4%)
Workplace isolation (works with)	
Alone	50 (35.0%)
With other podiatrists	41 (28.7%)
With other health professionals	19 (13.3%)
With other podiatrists and health professionals	33 (23.1%)
Patient caseload^a^	
General podiatry care^d^	103 (72.0%)
Musculoskeletal^e^	93 (65.0%)
High risk care^f^	78 (54.5%)
Rheumatological	22 (15.4%)
No patient caseload	3 (2.1%)
Daily patient contact^g^	
≤ 4 hours	11 (7.7%)
5–6 hours	42 (29.4%)
7–8 hours	66 (46.2%)
More than 8 hours	20 (14.0%)
Weekly work hours^h^	
Less than 10	6 (4.2%)
11–20	12 (8.4%)
21–30	17 (11.9%)
31–40	62 (43.4%)
41–45	28 (19.6%)
46–50	12 (8.4%)
More than 50	4 (2.8%)
Not currently working	2 (1.4%)

^a^ Multiple choice, total may exceed sample size.

^b^ For participants not in an ownership position.

^c^ For participants not currently enrolled in tertiary level education.

^d^ Comprises aged, skin and nail care.

^e^ Comprises biomechanics, neuro‐muscular pain, and orthotic prescription/manufacturing.

^f^ Comprises diabetes, wound, and vascular.

^g^ For participants with a patient caseload.

^h^ Percentages are presented relative to the total sample (*n* = 143) to provide a consistent denominator across all variables.

Workplace characteristics (Table 3) showed that over half of the participants reported heavy or excessive workloads, and approximately one‐quarter rated organisational support as poor or terrible. Around half reported satisfaction with remuneration and collegial support. Nearly two‐thirds reported high or very high stress levels, and most had experienced burnout at some point in their career. One third indicated they would not recommend podiatry to their younger self. Only 32.2% reported good or excellent supervision, with 48.3% reporting no supervision and the remaining 19.6% reporting average, poor or terrible supervision.

**TABLE 3:** Workplace and attitudinal characteristics (*n* = 143).

Variable	Summary statistics, *n* (%)
Workload	
Excessive	9 (6.3%)
Heavy	68 (47.6%)
Moderate	56 (39.2%)
Manageable	8 (5.6%)
Light	2 (1.4%)
Degree of supervisor support	
Terrible	5 (3.5%)
Poor	6 (4.2%)
Average	17 (11.9%)
Good	23 (16.1%)
Excellent	23 (16.1%)
No supervisor	69 (48.3%)
Degree of collegial support	
Terrible	2 (1.4%)
Poor	4 (2.8%)
Average	18 (12.6%)
Good	50 (35.0%)
Excellent	43 (30.1%)
No colleagues	26 (18.2%)
Degree of organisational support	
Terrible	10 (7.0%)
Poor	25 (17.5%)
Average	52 (36.4%)
Good	47 (32.9%)
Excellent	9 (6.3%)
Opportunity for professional development	
Terrible	11 (7.7%)
Poor	36 (25.2%)
Average	51 (35.7%)
Good	35 (24.5%)
Excellent	10 (7.0%)
Degree of work respect	
Extremely disrespected	2 (1.4%)
Somewhat disrespected	19 (13.3%)
Neither respected disrespected	21 (14.7%)
Somewhat respected	75 (52.4%)
Extremely respected	26 (18.2%)
Satisfaction with remuneration	
Very dissatisfied	12 (8.4%)
Somewhat dissatisfied	31 (21.7%)
Neither satisfied nor dissatisfied	28 (19.6%)
Somewhat satisfied	57 (39.9%)
Very satisfied	15 (10.5%)
Satisfaction with role	
Very dissatisfied	5 (3.5%)
Somewhat dissatisfied	15 (10.5%)
Neither satisfied nor dissatisfied	27 (18.9%)
Somewhat satisfied	64 (44.8%)
Very satisfied	32 (22.4%)
Stress	
Very high	15 (10.5%)
High	73 (51.0%)
Neither high nor low	44 (30.8%)
Low	11 (7.7%)
Very low	0 (0.0%)
Ever experienced burnout	
Yes	96 (67.1%)
No	35 (24.5%)
Unsure	12 (8.4%)
Recommend podiatry to younger self	
Yes	60 (42.0%)
No	48 (33.6%)
Unsure	35 (24.5%)

### 4.2 Objective i: Frequency of ITL

Table 4 presents the frequency of ITL, reasons for exit, and intended exit timeframes. Overall, 50.7% of podiatrists reported being highly or somewhat likely to leave the profession, as measured by the single‐item scale. The mean TIS‐6 score was 15.41 (SD 5.16). More than one‐fifth reported plans to exit within the next 2 years, and almost two‐fifths reported plans within 5 years. The most frequently self‐reported reasons for ITL were stress/burnout, dissatisfaction with remuneration, and limited professional development opportunities.

**TABLE 4:** Turnover intentions.

Variable	Summary statistics, *n* (%)
TIS1: How often have you considered leaving your job?	
Never	22 (12.8%)
Sometimes	68 (47.6%)
About half the time	25 (17.6%)
Most of the time	16 (10.9%)
Always	11 (8.0%)
TIS2: How satisfying is your job in fulfilling your personal needs?	
Totally dissatisfying	6 (4.3%)
Somewhat dissatisfying	20 (14.4%)
Neither satisfying or dissatisfying	26 (18.3%)
Somewhat satisfying	73 (51.4%)
Extremely satisfying	16 (11.6%)
TIS3: How often are you frustrated when not given the opportunity at work to achieve your personal work‐related goals?	
Never	30 (21.0%)
Sometimes	73 (51.7%)
About half the time	22 (15.2%)
Most of the time	14 (10.0%)
Always	3 (2.0%)
TIS4: How often do you dream about getting another job that will better suit your personal needs?	
Never	35 (24.9%)
Sometimes	52 (36.4%)
About half the time	20 (13.9%)
Most of the time	19 (13.3%)
Always	16 (11.3%)
TIS5: How likely are you to accept another job at the same compensation level should it be offered to you?	
Highly unlikely	40 (27.9%)
Somewhat unlikely	31 (21.8%)
Neither likely nor unlikely	30 (21.3%)
Somewhat likely	26 (18.0%)
Highly likely	15 (10.9%)
TIS6: How often do you look forward to another day at work?	
Never	21 (14.9%)
Sometimes	37 (25.9%)
About half the time	28 (19.7%)
Most of the time	50 (35.2%)
Always	6 (4.2%)
Total TIS:^c^	
Mean:	15.41
SD:	5.16
Low likelihood < 18	94 (66.3%)
High likelihood ≥ 18	48 (33.7%)
Likelihood of exit	
Highly unlikely	13 (9.0%)
Somewhat unlikely	26 (18.1%)
Neither likely nor unlikely	31 (21.6%)
Somewhat likely	27 (18.9%)
Highly likely	45 (31.8%)
Reasons for exit^a^ ^,^ ^b^ ^,^ ^d^	
Burnout/stress	43 (30.0%)
Dissatisfaction with remuneration	41 (28.6%)
Limited opportunity for professional development	36 (25.0%)
High workloads	34 (24.0%)
Reduced job satisfaction	31 (21.6%)
Retirement	31 (22.1%)
Overseas/other opportunities	25 (17.6%)
Lack of organisational support	23 (16.0%)
Underutilisation of knowledge/skillsets	20 (14.2%)
Family reasons	16 (10.9%)
Personal health reasons	14 (9.5%)
Lack of supervisor support	13 (9.2%)
Lack of collegial support	13 (9.2%)
Exit timeframe^a^ ^,^ ^d^	11 (7.5%)
6 months or less 6–12 months	14 (9.9%)
1–2 years	9 (6.4%)
2–5 years	21 (15.1%)
5–10 years	27 (18.8%)
More than 10 years	5 (3.8%)
Unsure	15 (10.8%)

Abbreviation: TIS = Turnover Intention Scale.

^a^ For participants with a neutral or likely exit likelihood.

^b^ Multiple choice, total may exceed sample size.

^c^ Maximum score of 30 (indicating high intention‐to‐leave).

^d^ Percentages are presented relative to the total sample for consistency (*n* = 143).

### 4.3 Factors Associated With ITL (Measured by the Single‐Item Likert Scale)

Full univariable results are presented in Table S1. The final four‐block hierarchical proportional‐odds ordinal logistic regression model examined factors associated with higher intentions of leaving (Table 5). Successive model blocks demonstrated incremental improvements in model performance, evident across model fit indices and explained variance estimates. The final model demonstrated the strongest overall exploratory performance.

**TABLE 5:** Hierarchical proportional‐odds ordinal logistic regression predicting greater ITL (measured by the single item Likert scale).

Predictor	Model A			Model B			Model C			Model D		
	OR	95% CI	*p*	OR	95% CI	*p*	OR	95% CI	*p*	OR	95% CI	*p*
Age^a^ (Years)												
26–30	0.86	0.18–3.84	0.85	1.00	0.20–4.56	1.00	1.49	0.30–7.11	0.62	0.93	0.18–4.58	0.93
31–35	0.48	0.08–2.77	0.41	0.33	0.05–1.95	0.22	0.44	0.07–2.63	0.37	0.44	0.07–2.57	0.37
36–40	0.24	0.03–1.65	0.15	0.27	0.04–1.91	0.19	0.40	0.06–2.74	0.35	0.33	0.05–2.31	0.27
41–45	0.40	0.044–3.82	0.42	0.41	0.05–4.02	0.44	0.36	0.04–0.06	0.35	0.34	0.04–2.87	0.33
46–50	0.08	0.01–0.87	0.040	0.06	0.01–0.74	0.029	0.07	0.01–0.73	0.027	0.05	0.00–0.60	0.019
51–55	0.18	0.02–1.75	0.14	0.21	0.02–2.10	0.19	0.28	0.03–2.83	0.28	0.24	0.02–2.36	0.23
56–60	1.20	0.11–13.51	0.89	1.30	0.12–14.92	0.83	1.87	0.17–20.88	0.61	1.53	0.13–17.25	0.73
≥ 61	1.21	0.10–14.70	0.88	0.74	0.06–9.42	0.81	0.65	0.05–7.82	0.74	0.90	0.07–11.09	0.94
Practice years^b^												
2–5	0.54	0.09–2.80	0.46	0.45	0.07–2.50	0.36	0.42	0.07–2.31	0.32	0.65	0.11–3.75	0.63
6–10	0.30	0.04–2.30	0.26	0.22	0.03–1.76	0.16	0.18	0.02–1.34	0.10	0.27	0.03–2.31	0.23
11–15	1.18	0.13–9.46	0.88	0.96	0.10–8.08	0.97	0.86	0.10–7.02	0.89	0.97	0.11–8.55	0.98
16–20	0.15	0.012–1.62	0.13	0.14	0.01–1.50	0.11	0.18	0.02–1.95	0.17	0.17	0.01–1.99	0.15
> 20	0.82	0.07–8.51	0.87	0.71	0.06–7.64	0.78	1.08	0.10–11.11	0.95	1.31	0.12–15.84	0.83
Patient contact^c^ (hours/day)												
5–6				1.03	0.29–3.73	0.96	1.24	0.32–4.82	0.76	0.73	0.17–3.09	0.67
7–8				1.03	0.30–3.51	0.96	0.89	0.24–3.27	0.86	0.58	0.15–2.31	0.44
> 8				4.83	1.12–21.91	0.036	5.86	1.28–28.53	0.025	3.30	0.66–17.45	0.15
Organisational support^d^							0.45	0.28–0.72	0.001	0.60	0.36–0.99	0.049
Stress^e^										0.57	0.35–0.92	0.023
Role satisfaction^d^										0.64	0.42–0.98	0.041
*N*	139											
Nagelkerke *R* ^2^	0.25			0.30			0.36			0.42		
Δ*R* ^2^	—			0.05			0.06			0.06		
*AIC*	377.12			374.36			365.32			357.32		
*LR χ* ^2^ (Δ)				*χ* ^2^(3) = 8.76			*χ* ^2^(1) = 11.04			*χ* ^2^(2) = 12.00		
*p*				0.033			< 0.001			0.002		

Abbreviations: AIC = Akaike Information Criterion; CI = confidence interval; LR *χ* ^2^ (Δ) = Likelihood Ratio chi‐squared test; Nagelkerke *R* ^2^ = estimate of explained variance; OR = odds ratio.

^a^ Entered as an ordered categorical predictor (reference category = 20–25 years).

^b^ Entered as a nominal variable (reference category < 2 years).

^c^ Entered as a nominal variable (reference category < 4 hours/day).

^d^ Ordinal variable coded with higher values indicating higher levels of the construct.

^e^ Ordinal variable coded with lower values indicating higher levels of the construct.

In the full adjusted model, lower organisational support, lower role satisfaction, and greater stress demonstrated the most consistent associations with greater ITL. Among demographic variables, podiatrists aged 46–50 years demonstrated lower odds of reporting greater ITL than the reference category; however, the CIs were wide, and the estimates should be interpreted cautiously. Other demographic and workload variables were retained as predefined covariates but were not independently associated with ITL.

In the data‐driven models, forward stepwise ordinal logistic regression identified a more parsimonious predictor set (Table S2). Age (particularly the 46–50‐year age group), organisational support, role satisfaction, and stress showed broadly similar associations across the primary hierarchical and alternative data‐driven models. Effect estimates varied across model specifications. Post‐stratification weighted analyses produced substantively equivalent findings; further details are provided in Supporting Information S2.

In summary, stronger leaving intentions on the single‐item measure were most clearly associated with lower organisational support, lower role satisfaction, and greater stress. Lower ITL was also observed among podiatrists aged 46–50 years, although the wide confidence interval warrants cautious interpretation.

### 4.4 Factors Associated With TIS‐6 Scores

Full bivariable results are presented in Table S3. A four‐block hierarchical linear regression model examined factors associated with TIS‐6 scores (Table 6). Successive model blocks demonstrated incremental improvements in model fit and explained variance across modelling stages. In the fully adjusted model, lower role satisfaction and lower perceived professional respect were associated with higher ITL scores. Fewer years in practice, lower household income contribution, and working in a non‐metropolitan region were also associated with higher ITL scores. Public sector employment showed a borderline association with higher ITL scores. Stress was not independently associated with the TIS‐6 scores in the fully adjusted model.

**TABLE 6:** Hierarchical multiple linear regression predicting higher TIS‐6 Scores.

Predictor	Model A			Model B			Model C			Model D		
	*B*	SE(*B*)	*β*	*B*	SE(*B*)	*β*	*B*	SE(*B*)	*β*	*B*	SE(*B*)	*β*
Practice years^a^	−0.47	0.31	−0.15	−0.46	0.30	−0.15	−0.60	0.27	−0.19*	−0.51	0.24	−0.16*
Household income contribution^a^	−1.13	0.59	−0.19	−1.35	0.57	−0.23*	−1.15	0.51	−0.19*	−1.10	0.47	−0.18*
Metro region^b^	−1.66	0.99	−0.15	−1.83	0.95	−0.17	−1.57	0.85	−0.15	−1.90	0.77	−0.18*
European ethnicity^c^	−3.12	1.24	−0.25*	−2.24	1.22	−0.18	−1.61	1.10	−0.13	−1.04	1.00	−0.08
Partnered^d^	−1.77	2.18	−0.08	−2.04	2.09	−0.09	−1.33	1.88	−0.06	−0.74	1.70	−0.03
Public sector^e^				3.96	1.24	0.23**	2.89	1.13	0.21*	2.08	1.05	0.15
Work respect^a^							−2.44	0.49	−0.41***	−1.14	0.51	−0.19*
Stress^f^										−0.92	0.53	−0.13
Role satisfaction^a^										−2.08	0.48	−0.38***
*N*	106											
*R* ^2^	0.18			0.25			0.41			0.53		
*Adjusted R* ^2^	0.14			0.21			0.36			0.48		
*AIC*	641.58			633.23			610.97			590.69		
*F* for change in *R* ^2^	4.31***			10.15**			25.20***			12.36***		

Abbreviations: *β* = standardized coefficient; AIC = Akaike Information Criterion; *B* = unstandardized coefficient; SE = standard error.

^a^ Ordinal variable with higher values indicating greater levels of the construct.

^b^ Entered as the nominal variable (reference category = works in non‐metropolitan region).

^c^ Entered as the nominal variable (Reference category = Māori, Asian, or other ethnicity).

^d^ Entered as the nominal variable (Reference category = non‐partnered).

^e^ Entered as the nominal variable (reference category = works in other sectors).

^f^ Ordinal variable coded with lower values indicating higher levels of the construct.

^*^ *p* *<* 0.05.

^**^ *p* *<* 0.01.

^***^ *p* ≤ 0.001.

In the data‐driven models, forward stepwise linear regression identified a more parsimonious predictor set (Table S4). Role satisfaction and perceived professional respect showed broadly similar associations across the primary hierarchical and alternative data‐driven models. Post‐stratification weighted analyses produced substantively equivalent findings; further details are provided in Supporting Information S2.

In summary, higher TIS‐6 scores were most clearly associated with lower role satisfaction and lower perceived professional respect. Higher scores were also associated with fewer years in practice, a lower contribution to household income, and working in a non‐metropolitan region.

## 5 Discussion

This study provides the first quantitative analysis of ITL among podiatrists in NZ. With over half of respondents reporting some ITL, these findings suggest a substantial risk of workforce attrition within a profession already characterised by limited growth and increasing service demand [8, 13]. A notable subgroup reported intentions to leave within the next 2 years, indicating both immediate and longer‐term retention challenges.

ITL levels appeared higher than those reported among rural Australian podiatrists, with approximately twice the proportion intending to leave within 5 years [11]. Although direct comparisons should be interpreted cautiously given differences in study populations and healthcare systems, structural distinctions between the workforces should also be considered. Australian podiatry is more integrated within publicly funded health services, providing broader access to funding and career opportunities [37]. Australian and UK podiatrists also have access to expanded scopes of practice, including scheduled medicines endorsement, which may offer greater opportunities for professional autonomy and career progression [38]. In contrast, NZ podiatry remains predominantly concentrated within small private‐practice settings, with significantly fewer publicly funded career pathways and formalised opportunities for clinical advancement [5]. Expanded scopes of practice, professional autonomy, and opportunities for career progression were not directly assessed in the present study; these structural characteristics are therefore presented to contextualise the findings rather than to explain the higher ITL observed among NZ podiatrists.

Compared with other NZ health professionals, the frequency of ITL in the present study also appeared high, exceeding that reported in the wider literature [16, 39]. Taken together, these comparisons suggest that NZ podiatry may experience workforce challenges that differ in nature or intensity from those experienced in other health professions and international podiatry settings.

Demographic and employment‐related factors showed less consistent relationships with ITL. Gender was not associated with leaving intentions, while patterns involving age and years in practice varied between ITL measures. A lower contribution to household income and working in a non‐metropolitan region were associated with higher TIS‐6 scores but not with the single‐item ITL measure. In contrast, workplace and psychosocial experiences showed greater consistency across the analyses. The adjusted single‐item ITL model identified role satisfaction, organisational support, and stress as the most salient factors. In the adjusted TIS‐6 model, lower role satisfaction and lower perceived professional respect were associated with higher ITL scores. Role satisfaction was therefore the only workplace factor associated with both ITL outcomes. Other associations observed in the preliminary analyses, including burnout, professional development opportunities, remuneration, and supervisory and collegial support, were attenuated after adjustment, suggesting that their contributions were less evident when other workplace and psychosocial factors were considered concurrently. Together, these findings suggest that ITL may emerge from interconnected workplace experiences rather than isolated factors, consistent with turnover theory and qualitative evidence among NZ health professionals [28, 40].

Respondents reporting lower role satisfaction also reported stronger leaving intentions across both ITL measures, making role satisfaction the factor most consistently associated with ITL. Within the broader literature, role satisfaction is conceptualised in relation to whether work is experienced as meaningful, valued, and sustainable [41, 42]; however, these dimensions were not assessed separately in the present study. This finding should be considered within the structural context of podiatry practice in NZ. Unlike professions such as physiotherapy, nursing, paramedicine, or medical imaging, which offer structured pathways into advanced clinical, specialist, and publicly funded roles, NZ podiatrists often remain concentrated within traditional practice settings, with fewer opportunities for diversification and career advancement [43, 44, 45, 46].

Although professional identity was not directly measured, lower perceived professional respect may contribute to reduced professional value and long‐term role sustainability [47]. Qualitative work among NZ podiatry students suggests that the profession may at times be perceived narrowly and negatively by students and other health professionals, potentially reinforcing perceptions of reduced professional value and limiting the profession's attractiveness as a long‐term career option [48].

Psychological strain was associated with a greater self‐reported ITL, consistent with evidence that strain may emerge within workplace contexts characterised by cumulative adversity [49, 50]. However, this association was not retained across all adjusted models, suggesting that strain interacts with broader workplace and psychosocial factors rather than operating independently [12].

These findings shift the focus of retention from increasing workforce supply alone to improving the sustainability of podiatry careers. The pattern of associations involving role satisfaction, organisational support, professional value, and psychological strain suggests that isolated initiatives may be insufficient to address the broader conditions associated with ITL. A coordinated approach across policy and workplace levels may therefore be warranted.

At a policy level, this may include clearer and more accessible pathways for career progression and diversification within NZ podiatry. Although expanded scopes of practice, such as designated prescribing rights or the introduction of an advanced practice scope, were not examined in the present study, evidence from physiotherapy in NZ indicates their potential to strengthen professional recognition and enable more varied career pathways [51, 52, 53]. Greater integration of podiatry within multidisciplinary and publicly funded services may similarly improve professional visibility and expand access to diverse clinical, funding and employment opportunities. These developments may be particularly relevant to supporting professional development and role satisfaction throughout podiatrists' careers [54].

At the workplace level, simply working alongside other practitioners was not associated with lower ITL, suggesting that proximity to colleagues alone may be insufficient to support retention. In contrast, supervisory and collegial support and professional development opportunities showed preliminary associations with lower ITL, although these were attenuated after adjustment. Structured supervision, mentorship, and accessible, high‐quality professional development may nevertheless represent plausible mechanisms for strengthening perceived organisational support, particularly for podiatrists working in small or isolated private practices. Hybrid mentorship and virtual peer‐support models may offer pragmatic approaches where in‐person support is not readily available. These strategies should be evaluated prospectively to determine whether they improve role sustainability and reduce subsequent attrition.

The methodological strengths of the study include the use of complementary outcome measures, a validated measure of turnover intention, and broadly consistent findings across multiple modelling approaches. However, several limitations should also be acknowledged. The cross‐sectional design precludes causal inference, and voluntary response sampling may introduce selection bias. Data were self‐reported and therefore subject to reporting bias. Although the sample was broadly representative, the predominance of practitioners working in private practice limited comparisons across employment settings. Some subgroup estimates were based on relatively small cell sizes, resulting in wide confidence intervals and reduced precision. Findings should therefore be interpreted as overall patterns of association rather than definitive subgroup‐specific effects.

Future longitudinal research is needed to determine the extent to which ITL translates into workforce attrition. Qualitative research exploring how podiatrists experience role satisfaction, professional value, and support may further clarify retention decisions. Further research should examine whether the association between role satisfaction and ITL varies by age, gender, or career stage and identify which retention initiatives are most relevant to newly qualified and younger podiatrists. Comparative research involving other allied health professions and international podiatry workforces may help determine whether factors associated with ITL differ in magnitude or influence across workforce contexts.

This study provides the first national quantitative assessment of ITL among NZ podiatrists. More than half of the respondents reported some ITL, highlighting a potential threat to workforce sustainability within an already capacity‐constrained workforce. ITL was consistently associated with modifiable workplace and psychosocial factors, particularly role satisfaction, organisational support, perceived professional respect, and psychological strain. Collectively, the findings suggest that leaving intentions arise through interconnected workforce experiences rather than isolated determinants. Retention initiatives should therefore extend beyond workforce supply strategies to address the broader organisational and professional environments in which podiatrists practice.

## Author Contributions

**Mia Clarke:** conceptualization (lead), methodology (lead), investigation (lead), data curation (lead), formal analysis (lead), funding acquisition (lead), project administration (lead), visualization (lead), writing – original draft (lead), writing – review and editing (equal). **Mike Frecklington:** conceptualization (lead), methodology (supporting), supervision (supporting), writing – review, and editing (equal). **Irene Zeng:** concepulization (supporting), methodology (supporting), formal analysis (supporting), validation (supporting), writing – review and editing (equal). **Matthew R. Carroll:** conceptualization (supporting), methodology (supporting), supervision (supporting), writing – review and editing (equal). **Richard J. Siegert:** conceptualization (supporting), methodology (supporting), supervision (supporting), writing – review and editing (equal). **Sarah Stewart:** conceptualization (lead), methodology (supporting), funding acquisition (supporting), project administration (supporting), visualization (supporting), supervision (lead), writing – review and editing (equal).

## Funding

This work was supported by Auckland University of Technology, New Zealand.

## Conflicts of Interest

This work was supported by the Auckland University of Technology Vice‐Chancellor's Doctoral Scholarship awarded to MC. The authors declare no other conflicts of interest.

## Supporting information


Supporting Information S1

Supporting Information S2

**Table S1**: Univariable ordinal logistic regression of factors associated with ITL (measured by the single item Likert scale).

**Table S2:** Stepwise ordinal logistic regression predicting greater ITL likelihood (measured by the single item Likert scale).

**Table S3:** Bivariable analyses of factors associated with TIS‐6 scores.

**Table S4:** Stepwise multiple linear regression predicting higher TIS‐6 scores.

## Data Availability

The data that support the findings of this study are available from the corresponding author upon reasonable request.
