# Supplementary material for: Intention‐to‐Leave Among New Zealand Podiatrists: A Cross‐Sectional Survey of Frequency and Associated Factors
Source: J Foot Ankle Res. 2026 Oct 21;19(4):e70236. doi: 10.1002/jfa2.70236 (PMC13687574; doi:10.1002/jfa2.70236)
Supplement: Supplementary file 1 [file JFA2-19-e70236-s001.docx]

| **Supplementary Table 2:** Stepwise ordinal logistic regression predicting greater ITL likelihood (measured by the single item Likert scale) | | | | | | |
| --- | --- | --- | --- | --- | --- | --- |
| **Predictor** | **B** | **SE** | **OR** | **95% CI** | **p** | **Relative importance (%)** |
| **Age^1^** |  |  |  |  |  | 38.65 |
| **26-30** | -.48 | .72 | .62 | .15 – 2.48 | .51 |  |
| **31-35** | -.91 | .75 | .40 | .09 – 1.74 | .23 |  |
| **36-40** | -1.29 | .71 | .28 | .07 – 1.09 | .07 |  |
| **41-45** | -1.10 | .81 | .33 | .06 – 1.62 | .18 |  |
| **46-50** | -2.58 | .83 | .08 | .01 - .37 | .002 |  |
| **51-55** | -.79 | .82 | .45 | .09 – 2.24 | .33 |  |
| **56-60** | .53 | .86 | 1.70 | .31- 9.35 | .54 |  |
| **≥61** | .36 | .86 | 1.43 | .26 – 7.99 | .68 |  |
| **Stress^a^** | -.47 | .25 | .63 | .38 – 1.02 | .06 | 5.60 |
| **Role satisfaction^b^** | -.43 | .21 | .65 | .43-.97 | .038 | 7.03 |
| **Patient contact (hours/day)^2^** |  |  |  |  |  | 21.24 |
| **5-6** | -1.10 | .72 | .33 | .08-1.37 | .13 |  |
| **7-8** | -.87 | .67 | .42 | .11-1.57 | .19 |  |
| **>8** | 1.03 | .80 | 2.79 | .59-13.70 | .20 |  |
| **Burnout^3^** |  |  |  |  |  | 12.44 |
| **No** | -.78 | .44 | .46 | .19-1.08 | .08 |  |
| **Unsure** | -1.60 | .65 | .20 | .05-.70 | .014 |  |
| **Organisational support^b^** | -.49 | .26 | .61 | .37-1.01 | .06 | 6.00 |
| **Other ethnicity^4^** | .97 | .55 | 2.63 | .93-7.94 | .08 | 5.30 |
| **General podiatry caseload^5^** | .63 | .42 | 1.88 | .83-4.35 | .13 | 3.72 |
| **Model statistics** | |  |  |  |  |  |
| **N** | 139 |  |  |  |  |  |
| **Nagelkerke R^2^** | .43 |  |  |  |  |  |
| **AIC** | 352.85 |  |  |  |  |  |
| ***LR*  χ² (Δ)** | χ²(18) = 72.09 | |  |  |  |  |
| ***p*** | <.001 |  |  |  |  |  |
| *B = log-odds coefficient; OR = odds ratio; CI = confidence interval; AIC = Akaike Information Criterion;* *LR χ² (Δ) =* ***Likelihood Ratio chi-squared test; Relative importance (%) reflects proportional contribution to model fit based on likelihood-ratio*** *χ²; Nagelkerke R² is reported as an estimate of explained variance; ¹Entered as an ordered categorical predictor (reference categrory = 20-25 years); ²Entered as a nominal variable (reference catergory < 2 years); ^3^Entered as a nominal variable (reference category = yes (burnout)); ⁴Entered as nominal variable (Reference category = Māori, NZ European, or Asian ethnicity); ⁵Entered as a nominal variable (Reference category = non-general podiatry caseload); ^a^Ordinal variable coded with lower values indicating higher levels of the construct; ^b^Ordinal variable coded with higher values indicating higher levels of the construct.* | | | | | | |
