# Supplementary material for: Intention‐to‐Leave Among New Zealand Podiatrists: A Cross‐Sectional Survey of Frequency and Associated Factors
Source: J Foot Ankle Res. 2026 Oct 21;19(4):e70236. doi: 10.1002/jfa2.70236 (PMC13687574; doi:10.1002/jfa2.70236)
Supplement: Supplementary file 2 [file JFA2-19-e70236-s002.docx]

| **Supplementary Table 3:** Bivariable analyses of factors associated with TIS-6 scores | | | | |
| --- | --- | --- | --- | --- |
| **Variable** | **Categories** | **Mean (SD)** | **Median (IQR)** | **P** |
| **Gender^1*^** | Male | 15.62 (4.89) | 15 (7) | .64^a^ |
|  | Female | 16.10 (5.40) | 15.00 (8.00) |  |
| **Age^2^** | 20-25 years | 17.85 (4.08) | 17.00 (7.00) | **<.001^b^** |
|  | 26-30 years | 18.30 (5.19) | 18.00 (9.00) |  |
|  | 31-35 years | 17.11 (4.94) | 18.00 (6.00) |  |
|  | 36-40 years | 15.50 (6.01) | 14.00 (9.00) |  |
|  | 41-45 years | 17.90 (5.88) | 18.00 (7.00) |  |
|  | 46-50 years | 16.38 (4.45) | 16.00 (7.00) |  |
|  | 51-55 years | 14.79 (4.54) | 14.00 (4.00) |  |
|  | 56-60 years | 13.45 (4.80) | 14.00 (10.00) |  |
|  | 61 years and over | 11.63 (3.26) | 11.00 (3.00) |  |
| **Ethnicity^1^** | NZ European | 15.18 (5.18) | 14.00 (7) | **.004^b^** |
|  | Non NZ European | 18.05 (4.89) | 18.00 (10) |  |
|  | Māori | 17.20 (5.61) | 17.00 (9) | .38^b^ |
|  | Non Māori | 15.87 (5.22) | 15.00 (7) |  |
|  | Asian | 17.93 (4.60) | 17.00 (8) | .11^b^ |
|  | Non Asian | 15.75 (5.20) | 15.00 (7) |  |
|  | Combined other | 18.00 (5.61) | 19.00 (11) | **.081^b^** |
|  | Non Combined other | 15.58 (5.10) | 15.00 (7) |  |
| **Marital status^3^** | Single (include divorced) | 15.31 (5.59) | 14.00 (7) | **.039^b^** |
|  | In a relationship | 18.53 (4.33) | 19.00 (7) |  |
|  | De facto partnership | 17.29 (4.51) | 17.00 (6) |  |
|  | Married | 15.23 (5.13) | 14.00 (8) |  |
| **Number of dependents^2^** | 0 | 16.28 (5.29) | 15.00 (7.00) | .26^b^ |
|  | 1 | 17.58 (4.78) | 17.00 (9.00) |  |
|  | 2 or more | 15.32 (5.26) | 14.00 (8.00) |  |
| **Household income contribution^2*^** | Less than 25% | 18.40 (4.30) | 18.50 (5.00) | **.040^b^** |
|  | 25-49% | 15.80 (4.64) | 15.00 (8.00) |  |
|  | 50%-74% | 15.17 (5.02) | 15.00 (8.00) |  |
|  | 75%-100% | 14.80 (5.95) | 13.00 (4.00) |  |
| **Region of practice^1^** | Wellington, Auckland, Canterbury | 16.57 (5.16) | 15.00 (7) | **.079^b^** |
|  | Combined other | 14.77 (5.25) | 14.50 (9) |  |
| **Practice years^2^** | Less than 2 years | 17.80 (5.05) | 19.00 (8.00) | **<.001^b^** |
|  | 2-5 years | 17.97 (4.50) | 18.00 (9.00) |  |
|  | 6-10 years | 14.94 (4.70) | 14.50 (8.00) |  |
|  | 11-15 years | 18.35 (6.53) | 19.00 (12.00) |  |
|  | 16-20 years | 14.82 (5.06) | 14.00 (5.00) |  |
|  | More than 20 years | 13.92 (4.44) | 13.00 (6.00) |  |
| **Sector^1^** | Private practice | 15.74 (5.09) | 15.00 (7.00) | .12^b^ |
|  | Non private practice | 19.00 (6.48) | 17.00 (12.00) |  |
|  | Public | 19.92 (5.54) | 20.00 (10.00) | **<.001^a^** |
|  | Non public | 15.13 (4.80) | 14.00 (6.00) |  |
|  | Combined other | 16.29 (5.74) | 15.00 (12.00) | .88^b^ |
|  | Non combined other | 15.95 (5.24) | 15.00 (7.00) |  |
|  | Mixed Sectors | 18.91 (5.36) | 19.50 (9.00) | **.004^a^** |
|  | Non Mixed Sectors | 15.43 (5.06) | 15.00 (7.00) |  |
| **Employment position^1^** | Owner/co-owner | 15.01 (5.55) | 14.00 (7.00) | **.018^b^** |
|  | Non owner/co-owner | 16.80 (4.84) | 16.50 (7.00) |  |
|  | Permanent employee | 17.33 (4.97) | 18.00 (6.00) | **.008^a^** |
|  | Non-permanent | 14.98 (5.24) | 14.00 (8.00) |  |
|  | Combined other | 15.94 (5.65) | 14.00 (9.00) | .72^b^ |
|  | Non combined other | 15.97 (5.14) | 15.00 (7.00) |  |
| **Future ownership intent^2*^** | Definitely not | 16.50 (5.35) | 16.50 (10.00) | **.012^b^** |
|  | Probably not | 14.95 (4.34) | 15.00 (5.00) |  |
|  | Might or might not | 16.71 (4.34) | 18.00 (5.00) |  |
|  | Probably yes | 18.25 (3.96) | 18.5 (6.00) |  |
|  | Definitely yes | 19.60 (5.48) | 21.0 (6.00) |  |
| **Secondary employment outside of podiatry^1^** | Yes | 17.50 (4.63) | 18.0 (6.00) | .21^a^ |
|  | No | 15.77 (5.30) | 15.0 (7.00) |  |
| **Education^1^** | Diploma/Bachelors | 16.15 (6.32) | 16.5 (8) | .45^b^ |
|  | Postgraduate | 15.68 (5.17) | 14.0 (6) |  |
| **Current enrolment^1^** | Yes | 18.50 (4.94) | 17.5 (7) | **.050^b^** |
|  | No | 15.69 (5.22) | 15.0 (7) |  |
| **Future enrolment intent^3*^** | Yes | 16.03 (5.22) | 14.5 (7) | .38^a^ |
|  | No | 14.96 (5.57) | 14.0 (7) |  |
|  | Unsure | 16.38 (4.73) | 15.5 (7) |  |
| **Workplace isolation^3^** | Alone | 16.24 (5.24) | 16.0 (8) | .79^b^ |
|  | With other podiatrists | 15.24 (4.84) | 15.0 (7) |  |
|  | With other health professionals | 15.84 (5.10) | 14.0 (6) |  |
|  | With other podiatrists and health professionals | 16.52 (5.91) | 15.0 (8) |  |
| **Patient caseload^1^** | General podiatry care | 16.11 (4.98) | 15.0 (8) | .60^a^ |
|  | No General podiatry care | 15.60 (5.91) | 14.0 (8) |  |
|  | Musculoskeletal | 15.14 (4.83) | 14.0 (7) | **.010^a^** |
|  | No Musculoskeletal | 17.50 (5.67) | 16.0 (9) |  |
|  | Diabetes, wound, vascular | 16.31 (5.54) | 15.0 (7) | .46^b^ |
|  | No diabetes, wound, vascular | 15.55 (4.87) | 15.0 (8) |  |
|  | Rheumatological | 14.36 (5.41) | 13.5 (5) | **.088^b^** |
|  | No Rheumatological | 16.26 (5.18) | 15.0 (8) |  |
|  | Mixed caseload | 16.27 (5.33) | 15.0 (8) | .32^a^ |
|  | No mixed caseload | 15.35 (5.06) | 15.0 (7) |  |
| **Daily patient contact^2*^** | Less than 4 hours | 14.18 (4.79) | 12.0 (8) | .69^b^ |
|  | 5-6 hours | 16.60 (5.07) | 15.5 (7) |  |
|  | 7-8 hours | 16.17 (5.52) | 15.0 (8) |  |
|  | More than 8 hours | 14.55 (4.89) | 14.0 (8) |  |
| **Weekly work hours^2^** | Less than 20 hours | 15.60 (4.78) | 15.0 (9) | .36^a^ |
|  | 21-30 hours | 15.71 (5.12) | 15.0 (7) |  |
|  | 31-40 hours | 15.97 (5.41) | 15.0 (8) |  |
|  | 41-45 hours | 15.50 (4.59) | 14.5 (7) |  |
|  | 46 hours or more | 17.50 (6.56) | 15.0 (8) |  |
| **Workload^2^** | Excessive/Heavy | 16.32 (5.40) | 16.0 (7) | .50^b^ |
|  | Moderate | 15.25 (5.07) | 14.0 (7) |  |
|  | Manageable/Light | 17.20 (4.89) | 17.5 (9) |  |
| **Degree of supervisor support^3^** | Terrible/Poor | 22.00 (4.94) | 24.0 (7) | **<.001^a^** |
|  | Average | 19.41 (5.15) | 20.0 (11) |  |
|  | Good | 17.39 (3.64) | 18.0 (4) |  |
|  | Excellent | 13.78 (4.62) | 13.0 (6) |  |
|  | No supervisor | 14.41 (4.77) | 14.0 (6) |  |
| **Degree of collegial support^3^** | Average/Poor/Terrible | 21.00 (4.50) | 20.5 (6) | **<.001^a^** |
|  | Good | 15.80 (4.66) | 15.0 (6) |  |
|  | Excellent | 13.67 (4.72) | 13.0 (6) |  |
|  | No colleagues | 15.42 (4.88) | 15.5 (7) |  |
| **Degree of organisational support^2^** | Terrible/Poor | 18.60 (5.05) | 18.0 (10) | **<.001^a^** |
|  | Average | 16.08 (5.07) | 15.0 (8) |  |
|  | Good/Excellent | 14.21 (4.88) | 14.0 (8) |  |
| **Opportunity for professional development^2^** | Terrible/Poor | 18.55 (5.35) | 18.0 (10) | **<.001^a^** |
|  | Average | 15.16 (5.61) | 14.0 (7) |  |
|  | Good/Excellent | 14.18 (4.85) | 14.0 (7) |  |
| **Degree of work respect^2^** | Extremely/Somewhat disrespected | 20.62 (4.88) | 20.0 (7) | **<.001^a^** |
|  | Neither respected disrespected | 19.29 (5.33) | 19.0 (9) |  |
|  | Somewhat respected | 15.05 (3.94) | 14.0 (6) |  |
|  | Extremely respected | 12.15 (4.84) | 11.5 (5) |  |
| **Satisfaction with remuneration^2^** | Very dissatisfied | 22.50 (3.78) | 23.0 (6) | **<.001^b^** |
|  | Somewhat dissatisfied | 18.39 (4.94) | 18.0 (7) |  |
|  | Neither satisfied nor dissatisfied | 15.46 (4.32) | 15.0 (6) |  |
|  | Somewhat satisfied | 14.53 (4.19) | 14.0 (7) |  |
|  | Very satisfied | 12.13 (5.89) | 10.0 (7) |  |
| **Satisfaction with role^2^** | Very/somewhat dissatisfied | 23.10 (3.80) | 23.50 (6) | **<.001^b^** |
|  | Neither satisfied nor dissatisfied | 17.78 (3.89) | 18.0 (6) |  |
|  | Somewhat satisfied | 14.94 (4.02) | 15.0 (6) |  |
|  | Very satisfied | 12.03 (4.20) | 12.0 (5) |  |
| **Stress^2^** | Very high | 20.13 (5.98) | 20.0 (10) | **<.001^b^** |
|  | High | 16.29 (5.23) | 16.0 (7) |  |
|  | Neither high nor low | 14.55 (4.16) | 14.0 (4) |  |
|  | Low | 13.82 (5.31) | 13.0 (9) |  |
| **Burnout experience^3^** | Yes | 16.73 (5.27) | 15.0 (8) | **.037^b^** |
|  | No | 13.97 (4.29) | 14.0 (7) |  |
|  | Unsure | 15.67 (6.33) | 15.0 (11) |  |
| **Recommend podiatry^3^** | Yes | 12.52 (3.56) | 12.0 (5) | **<.001^a^** |
|  | No | 19.29 (5.05) | 19.0 (10) |  |
|  | Unsure | 17.31 (4.37) | 18.0 (7) |  |
| **Likelihood of exit^2^** | Highly unlikely | 10.67 (2.77) | 11.0 (5) | **<.001^b^** |
|  | Somewhat unlikely | 13.50 (3.85) | 13.0 (3) |  |
|  | Neither likely nor unlikely | 15.31 (3.12) | 15.0 (5) |  |
|  | Somewhat likely | 17.72 (4.97) | 18.0 (6) |  |
|  | Highly likely | 17.86 (6.29) | 19.0 (11) |  |
| **Reasons for exit^1*^** | High workloads | 18.75 (5.02) | 16.50 (7.00) | **<.001^a^** |
|  | No high workloads | 14.88 (4.94) | 14.00 (7.00) |  |
|  | Lack of supervisor support | 21.00 (4.32) | 20.50 (6.00) | **<.001^a^** |
|  | No lack of supervisor support | 15.33 (5.01) | 14.00 (7.00) |  |
|  | Lack of collegial support | 19.13 (5.46) | 18.00 (8.00) | **.021^b^** |
|  | No lack of collegial support | 15.59 (5.11) | 15.00 (7.00) |  |
|  | Lack of organisational support | 19.52 (5.12) | 18.00 (9.00) | **<.001^b^** |
|  | No lack of organisational support | 15.06 (4.90) | 14.00 (8.00) |  |
|  | Underutilisation of knowledge/skillsets | 20.60 (5.30) | 22.00 (8.00) | **<.001^b^** |
|  | No underutilisation of knowledge/skillsets | 14.98 (4.70) | 14.00 (6.00) |  |
|  | Limited opportunity for professional development | 19.73 (4.33) | 20.00 (8.00) | **<.001^a^** |
|  | No limited opportunity for professional development | 14.29 (4.73) | 14.00 (7.00) |  |
|  | Dissatisfaction with renumeration | 18.71 (4.35) | 19.00 (7.00) | **<.001^a^** |
|  | No dissatisfaction with renumeration | 14.70 (5.15) | 14.00 (7.00) |  |
|  | Burnout/stress | 18.60 (4.82) | 18.50 (7.00) | **<.001^a^** |
|  | No burnout/stress | 14.55 (4.92) | 14.00 (7.00) |  |
|  | Reduced job satisfaction | 18.68 (5.16) | 19.00 (7.00) | **<.001^a^** |
|  | No reduced job satisfaction | 15.12 (5.00) | 14.00 (7.00) |  |
|  | Personal health reasons | 17.93 (4.13) | 17.00 (5.00) | **.065^b^** |
|  | No personal health reasons | 15.73 (5.32) | 14.5 (7) |  |
|  | Family reasons | 17.12 (4.73) | 16.0 (8) | .31^b^ |
|  | No family reasons | 15.81 (5.31) | 15.0 (7) |  |
|  | Retirement | 12.42 (4.14) | 11.5 (5) | **<.001^b^** |
|  | No retirement | 16.75 (5.15) | 16.0 (7) |  |
|  | Overseas/other opportunities | 19.03 (4.48) | 19.0 (8) | **<.001^a^** |
|  | No overseas/other opportunities | 15.15 (5.15) | 14.0 (7) |  |
| **Exit timeframe^3*^** | Less than 12 months | 18.30 (6.63) | 18.0 (10) | **.028^a^** |
|  | 1-5 years | 18.48 (4.93) | 19.0 (8) |  |
|  | 5-10 years | 15.08 (4.04) | 15.0 (6) |  |
|  | Unsure | 16.65 (4.53) | 15.5 (7) |  |
| *^a^P-value from parametric test; ^b^P-value from non-parametric test; Bolded P values indicate significance at <0.10.^1^Dichotomous variable (two-tailed independent samples t-tests (or non-parametric Mann-Whitney U tests)); ^2^Ordinal variable (ANOVA with trend analysis (or non-parametric Jonckheere-Terpstra test)); ^3^Nominal variable (ANOVA) (or non-parametric Kruskal-Wallis H tests); *missing data due to skip logic or category collapsing/removal for regression analyses.* | | | | |
