# Supplementary material for: Intention‐to‐Leave Among New Zealand Podiatrists: A Cross‐Sectional Survey of Frequency and Associated Factors
Source: J Foot Ankle Res. 2026 Oct 21;19(4):e70236. doi: 10.1002/jfa2.70236 (PMC13687574; doi:10.1002/jfa2.70236)
Supplement: Supplementary file 3 [file JFA2-19-e70236-s003.docx]

| **Supplementary Table 4:** Stepwise multiple linear regression predicting higher TIS-6 scores | | | | | | |
| --- | --- | --- | --- | --- | --- | --- |
| **Predictor** | **B** | **SE** | **β** | **95% CI** | **p** | **LMG %** |
| **Role satisfaction^1^** | -2.08 | .43 | -.39 | -2.93, -1.23 | <.001 | 35.2 |
| **Respect^1^** | -1.16 | .44 | -.21 | -2.03, -.28 | .010 | 24.7 |
| **Collegial support^1^** | -1.34 | .50 | -.19 | -2.32, -.35 | .008 | 16.7 |
| **Professional development^1^** | -.93 | .45 | -.14 | -1.82, -.03 | .043 | 11.1 |
| **Permanent employment position^2^** | 2.18 | .66 | .41 | .87, 3.49 | .001 | 7.8 |
| **Metropolitan region^3^** | -2.12 | .71 | -.40 | -3.52, -.72 | .003 | 4.5 |
| **Model statistics** | |  |  |  |  |  |
| **N** | 117 |  |  |  |  |  |
| **R^2^** | .59 |  |  |  |  |  |
| **Adjusted R^2^** | .57 |  |  |  |  |  |
| **AIC** | 634.81 |  |  |  |  |  |
| **Residual SE** | 3.51 |  |  |  |  |  |
| ***F*** | (6,110) = 26.20 | |  |  |  |  |
| ***p*** | <.001 |  |  |  |  |  |
| *B = Unstandardized coefficients; SE = Standard Errors; β = standardized coefficient; CI = confidence interval; AIC = Akaike Information Criterion; LMG = Lindeman–Merenda–Gold relative importance metric, representing each predictor’s averaged contribution to R²; ^1^Entered as as ordinal variables with numerically with higher scores indicating greater degree of the construct; ^2^Entered as nominal variable (reference category = has other employment position); ^3^Entered as nominal variable (reference category = works in non-metropolitan region); ^4^Coded as ordinal variable with lower numerical indicating higher levels of the construct; *reflects the combine contribution of the polynomical terms.* | | | | | | |
