# Supplementary material for: Intention‐to‐Leave Among New Zealand Podiatrists: A Cross‐Sectional Survey of Frequency and Associated Factors
Source: J Foot Ankle Res. 2026 Oct 21;19(4):e70236. doi: 10.1002/jfa2.70236 (PMC13687574; doi:10.1002/jfa2.70236)
Supplement: Supplementary file 4 [file JFA2-19-e70236-s004.docx]

**Supporting Information 1:** Online Survey

Section 1: Demographic information:

1. What gender do you identify as?

- Male
- Female
- Another Gender

2. What is your age?

- 20-25 years
- 26-30 years
- 31-35 years
- 36-40 years
- 46-50 years
- 51-60 years
- 61-65 years
- 66 years or older

3. What race or ethnic group best describes you? *(you may select more than one answer)*

- European
- Māori
- What is your iwi/hapu? _______
- Pacific
- Asian
- Middle Eastern, Latin American or African
- Other Ethnicity

4. What region/location do you primarily practice in?

- Northland
- Auckland
- Waikato
- Bay of Plenty
- Gisborne
- Hawkes Bay
- Taranaki
- Manawatu-Wanganui
- Wellington
- Marlborough
- Nelson
- Tasman
- West Coast
- Canterbury
- Otago
- Southland
- Chatham Islands

5. What is your martial status?

- Single
- In a relationship
- Married
- Divorced/separated
- Widowed
- Prefer not to say

6. How many children/dependents do you have?

- None
- 1
- 2-3
- 4 or more

6.1 What is your percentage contribution to your household income? *(if has children/dependents)*

- Less than 25%
- 25%-49%
- 50%-74%
- 75%-100%

7. How many years have you practiced as a podiatrist?

- Less than 2 years
- 2-5 years
- 6-10 years
- 11-15 years
- 16-20 years
- More than 20 years

Section 2: Job context

8. Which podiatry sector(s) are you currently employed in? *(you may select more than one answer)*

- Private practice
- Public
- Research
- Education
- Other
- Please specify: _________

9. What is your current employment position(s) as a podiatrist? *(you may select more than one answer)*

- Owner/co-owner
- Permanent employee
- Fixed-term/casual employee
- Independent contractor
- Other
- Please specify: _______

9.1. Would you like to own/co-own a podiatry practice in the future? (if currently not an owner/co-owner)

- Definitely not
- Probably not
- Might or might not
- Probably yes
- Definitely yes

10. Do you have other employment outside of the podiatry profession?

- Yes
- No

11. What is the highest level of formal education you have completed?

- Diploma
- Bachelor’s degree
- Postgraduate certificate/diploma/bachelor’s honours degree
- Master’s degree
- Doctoral degree

12. Are you currently enrolled in university study or other formal qualifications?

- Yes
- No

12.1. Are you planning to enrol in university study or other formal qualifications in the future*? (if not currently enrolled)*

- Yes
- No
- Unsure

13. Which of the following statements best describes your daily workplace?

- I work by myself most of the time
- I work with other podiatrists most of the time
- I work with other health professionals most of the time
- I work with other health professionals and other podiatrists most of the time

14. What best describes your patient caseload? *(you may select more than one answer)*

- Dermatological care
- Musculoskeletal care
- Diabetic care
- Rheumatological care
- Other speciality
  - Please specify: ______
- I do not have a patient caseload

15. On average, how many hours of patient contact do you have per working day?

- 0
- 1-2
- 3-4
- 5-6
- 7-8

16. On average, how many hours do you work a week?

- Not currently working *(maternity leave, LWOP, sabbatical, between jobs etc)*
- Less than 10 hours
- 10-20 hours
- 21-30 hours
- 31-40 hours
- 41-50 hours
- More than 50 hours

Section 3: Workplace climate

17. How would you describe your day-to-day workload?

- Excessive (constantly too much)
- Heavy (often overwhelming)
- Moderate (some busy days)
- Manageable (steady pace)
- Light (rarely busy)

18. How would you describe the level of support from your supervisor?

- Terrible
- Poor
- Average
- Good
- Excellent
- I do not have a supervisor in my job

19. How would you describe the level of support from your colleagues?

- Terrible
- Poor
- Average
- Good
- Excellent
- I do not have a supervisor in my job

20. How would you describe the level of support from the wider podiatry organisation?

- Terrible
- Poor
- Average
- Good
- Excellent

21. How would you describe your satisfaction with your remuneration/work benefits?

- Very dissatisfied
- Somewhat dissatisfied
- Neither satisfied nor dissatisfied
- Somewhat satisfied
- Very satisfied

22. How satisfied are you in your daily role as a podiatrist?

- Very dissatisfied
- Somewhat dissatisfied
- Neither satisfied nor dissatisfied
- Somewhat satisfied
- Very satisfied

23. How respected do you feel in your work?

- Extremely disrespected
- Somewhat disrespected
- Neither respected nor disrespected
- Somewhat respected
- Extremely respected

25. How would you describe your stress levels in your work?

- Very high
- High
- Neither high nor low
- Low
- Very low

26. Have you ever experienced 'burnout' or significant unmanageable stress from your work?

- Yes
- No
- Unsure

27. Would you recommend podiatry as a career to your younger self?

- Yes
- No
- Unsure

Section 4: Turnover intentions

Turnover Intention Scale

The following section aims to ascertain the extent to which you intend to say in the NZ podiatry profession

TIS 1: How often have you considered leaving the NZ podiatry profession?

- Never
- Sometimes
- About half the time
- Most of the time
- Always

TIS 2: How satisfying is your job in fulfilling your personal needs?

- Totally dissatisfying
- Somewhat dissatisfying
- Neither satisfying nor dissatisfying
- Somewhat satisfying
- Extremely satisfying

TIS 3: How often are you frustrated when not given the opportunity at work to achieve your personal work-related goals?

- Never
- Sometimes
- About half the time
- Most of the time
- Always

TIS 4: How often do you dream about getting another job that will better suit your personal needs?

- Never
- Sometimes
- About half the time
- Most of the time
- Always

TIS 5: How likely are you to accept another job at the same compensation level should it be offered to you?

- Highly unlikely
- Somewhat unlikely
- Neither likely nor unlikely
- Somewhat likely
- Highly likely

TIS 6 How often do you look forward to another day at work?

- Never
- Sometimes
- About half the time
- Most of the time
- Always

What is the likelihood of you leaving the NZ podiatry profession in the future?

- Highly unlikely
- Somewhat unlikely
- Neither likely nor unlikely
- Somewhat likely
- Highly likely

What are the main reasons for driving this likelihood? *(you may select more than one answer)*

- High workloads
- Lack of supervisor support
- Lack of collegial support
- Lack of organisational support
- Underutilisation of knowledge/skillsets
- Limited opportunity for professional growth/development
- Dissatisfaction with remuneration or work benefits
- Burnout/stress
- Reduced job satisfaction
- Personal health reasons
- Family reasons
- Retirement
- Overseas opportunities
- Other
  - Please specify_____

What time frame do you expect to leave the profession within?

- 6 months or less
- 6-12 months
- 1-2 years
- 2-5 years
- 5-10 years
- More than 10 years
- Unsure
