# Supplementary material for: Intention‐to‐Leave Among New Zealand Podiatrists: A Cross‐Sectional Survey of Frequency and Associated Factors
Source: J Foot Ankle Res. 2026 Oct 21;19(4):e70236. doi: 10.1002/jfa2.70236 (PMC13687574; doi:10.1002/jfa2.70236)
Supplement: Supplementary file 5 [file JFA2-19-e70236-s005.docx]

**Supporting Information 2:** Model sensitivity and assumption checks

**2.1:** Logistic regression models

Model diagnostics indicated that assumptions for the hierarchical approach were largely satisfied. The proportional-odds assumption was supported by a non-significant global Brant test for the final model (χ²(38) = 22.23, p = 1.00). Minor predictor-level deviations were observed but were not considered to materially affect model validity. Tests of nominal effects were non-significant, and multicollinearity was low (GVIF range 1.05 to 1.17). Overall, the final model demonstrated stable estimation. Diagnostics for the stepwise model were similarly acceptable, with no evidence of major assumption violations or model instability. The proportional-odds assumption was also supported by a non-significant global Brant Test (χ²(36) = 11.8, p = 1.00), and multicollinearity remained low (GVIF range 1.05 to 1.17).

**2.2:** Linear regression models

Model diagnostics indicated that assumptions for the hierarchical linear regression were satisfied. Multicollinearity was low (GVIF range 1.04-1.52), and influence diagnostics identified no problematic outliers (maximum Cook’s D = .12). Residual diagnostics supported the assumptions of normality, linearity, and homoscedasticity. Sensitivity analyses demonstrated that alternative coding of demographic variables did not materially affect model estimates, and more parsimonious specifications were therefore retained. For ordinal predictors, inspection of partial regression plots supported approximate linearity, and numeric coding was retained as dummy-coded alternatives did not improve model fit or reduce AIC. Inclusion of alternative workplace climate or strain indicators (e.g., organisational support; burnout) did not meaningfully improve explained variance or alter substantive conclusions.

Diagnostics for the stepwise model were similarly acceptable, with no evidence of major violations of linear model assumptions. Multicollinearity was low (GVIF range 1.03 to 1.77), and the Breusch-Pagan test did not indicate significant heteroskedasticity (BP = 10.86, p = .093). Residual plots showed no major deviations from model assumptions, and sensitivity analyses using alternative predictor specifications yielded comparable model performance and consistent substantive inferences.

**2.3 Post-stratification weighted analyses**

Compared with the NZ podiatry workforce, the survey sample included a higher proportion of female respondents (73.4% vs 66%) and modest differences in age distribution, with younger podiatrists slightly overrepresented. To assess the potential influence of these differences, post-stratification weights based on workforce age and sex distributions were applied to the hierarchical regression models. Weighted analyses produced substantially equivalent results to the primary analyses for both ITL outcomes, indicating that the observed associations were robust to potential sampling and non-response bias.
