# Supplementary material for: Intention‐to‐Leave Among New Zealand Podiatrists: A Cross‐Sectional Survey of Frequency and Associated Factors
Source: J Foot Ankle Res. 2026 Oct 21;19(4):e70236. doi: 10.1002/jfa2.70236 (PMC13687574; doi:10.1002/jfa2.70236)
Supplement: Supplementary file 6 [file JFA2-19-e70236-s006.docx]

| **Supplementary Table 1.** Univariable ordinal logistic regression of factors associated with ITL (measured by the single item Likert scale) | | | | |
| --- | --- | --- | --- | --- |
| **Predictor** | **Level/Contrast** | **OR (95% CI)** | **LR p-value** | **FDR (LR)** |
| **Gender^a^** | Female vs Males | 1.27 (.64 – 2.54) | .49 | .67 |
| **Age^b^** | 26-30 vs 20-25 | .61 (.17 – 2.18) | **<.001** | .013 |
|  | 31-35 vs 20-25 | .35 (.09 – 1.32) |  |  |
|  | 36-40 vs 20-25 | .18 (.05 - .67) |  |  |
|  | 41-45 vs 20-25 | .34 (.08 – 1.49) |  |  |
|  | 46-50 vs 20-25 | .09 (.02 - .37) |  |  |
|  | 51-55 vs 20-25 | .19 (.05 - .78) |  |  |
|  | 56-60 vs 20-25 | 1.02 (.23 – 4.52) |  |  |
|  | 61+ vs 20-25 | 1.29 (.30 – 5.65) |  |  |
| **NZ/Euro ethnicity^a^** | NZ Euro vs Non NZ Euro | .60 (.31 – 1.16) | **.128** | .27 |
| **Māori ethnicity^a^** | Māori vs Non Māori | .88 (.27 – 2.87) | .84 | .88 |
| **Asian ethnicity^a^** | Asian vs Non Asian | 1.11 (.44 – 2.80) | .83 | .88 |
| **Other ethnicity^a^** | Other vs Non-other | 2.51 (1.07 – 5.87) | **.031** | .098 |
| **Partnered^a^** | Partnered vs Non partnered | 1.27 (.59 – 2.73) | .54 | .72 |
| **Number of dependents^c^** | Per 1-unit increase | .610 (.43 - .82) | **.001** | .014 |
| **Household income contribution^b^** | 25% - 49% vs < 25% | .27 (.07 – 1.00) | **.146** | .29 |
|  | 50% - 74% vs <25% | .44 (.12 – 1.53) |  |  |
|  | 75% - 100% vs <25% | .24 (.06 – 1.01) |  |  |
| **Region of practice^a^** | Non metro Vs Metro | 1.19 (.63 – 2.24) | .59 | .74 |
| **Practice years^a^** | Per 1-unit increase | .89 (.76 – 1.06) | **.187** | .34 |
| **Private sector^a^** | Private vs Non private | .76 (.25 – 2.32) | .62 | .74 |
| **Public sector^a^** | Public vs Non public | 1.10 (.50 – 2.40) | .82 | .88 |
| **Other sector^a^** | Other vs Non other | .39 (.10 – 1.55) | **.175** | .34 |
| **Mixed sectors^a^** | Mixed vs Non mixed | .74 (.32 – 1.69) | .47 | .66 |
| **Employment owner/co-owner^a^** | Owner vs Non owner | .61 (.34 – 1.11) | **.103** | .25 |
| **Employment permanent^a^** | Permanent vs Non-permanent | 1.48 (.81 – 2.71) | **.199** | .35 |
| **Employment other^a^** | Other vs Non other | .95 (.47 – 1.92) | .89 | .91 |
| **Secondary employment outside of podiatry^a^** | Yes vs No | 1.05 (.42 – 2.63) | .91 | .91 |
| **Highest education^a^** | Postgraduate vs Undergraduate | .69 (.38 – 1.27) | .23 | .38 |
| **Current enrolment^a^** | Yes vs No | 2.28 (.85 – 6.11) | **.096** | .25 |
| **Future enrolment intent^a^** | No vs Yes | 1.56 (.70 – 3.48) | **.006** | .045 |
|  | Unsure vs Yes | 0.48 (.21 – 1.10) |  |  |
| **Workplace isolation^a^** | Other podiatrists vs self | .73 (.34 – 1.57) | .28 | .44 |
|  | Other health pros vs self | .45 (.18 – 1.15) |  |  |
|  | Both vs self | 0.54 (.25 – 1.19) |  |  |
| **General pod caseload^a^** | Yes vs No | 1.81 (.91 – 3.59) | **.088** | .24 |
| **MSK caseload^a^** | Yes vs No | .83 (.45 – 1.55) | .56 | .72 |
| **DM/wound caseload^a^** | Yes vs No | 1.60 (.88 – 2.90) | **.122** | .27 |
| **Rheum caseload^a^** | Yes vs No | .86 (.36 – 2.07) | .74 | .84 |
| **Mixed caseload^a^** | Mixed vs Non Mixed | 2.01 (1.06 – 3.79) | **.031** | .098 |
| **Daily patient contact^b^** | 5-6 hours vs <4 hours | 1.05 (.33 – 3.67) | **.027** | .098 |
|  | 7-8 hours vs <4 hours | 1.22 (.40 – 3.72) |  |  |
|  | >8 hours vs <4 hours | 4.47 (1.15 – 17.31). |  |  |
| **Weekly work hours^b^** | 21-30 hours vs <20 | 1.40 (.43 – 4.50) | .34 | .52 |
|  | 31-40 hours vs <20 | 1.33 (.06 – 3.19) |  |  |
|  | 41-45 hours vs <20 | .62 (.23 – 1.66) |  |  |
|  | ≥ 46 hours vs <20 | 1.52 (.46 – 5.07) |  |  |
| **Workload^c^** | Per 1-unit increase | .82 (.52 – 1.30) | .41 | .60 |
| **Supervisor support^a^** | No vs Yes | .86 (.48 – 1.56) | .62 | .74 |
| **Level of supervisor support^c^** | Per 1-unit increase | .60 (.39 - .93) | **.020** | .090 |
| **Collegial support^a^** | No vs Yes | .88 (.41 – 1.89) | .75 | .84 |
| **Level of collegial support^c^** | Per 1-unit increase | .61 (.39 - .96) | **.030** | .098 |
| **Degree of organisational support^c^** | Per 1-unit increase | .51 (.35 - .76) | **<.001** | .013 |
| **Opportunity for professional development^c^** | Per 1-unit increase | .65 (.45 - .94) | **.020** | .090 |
| **Degree of work respect^c^** | Per 1-unit increase | .74 (.53 – 1.03) | **.072** | .210 |
| **Satisfaction with remuneration^c^** | Per 1-unit increase | .70 (.53 - .92) | **.009** | .054 |
| **Satisfaction with role^c^** | Per 1-unit increase | .60 (.42 - .84) | **.003** | .023 |
| **Stress^c^** | Per 1-unit increase | .50 (.33 - .76) | **<.001** | .013 |
| **Burnout experience^a^** | No vs Yes | .49 (.24 - .99) | **.013** | .070 |
|  | Unsure vs Yes | .26 (.09 - .74) | **.013** | .070 |
| *OR = odds ratio; CI = confidence intervals (Wald estimates from the fitted models); LR = likelihood‑ratio; FDR = false discovery rate; Bold LR p-values indicate variables meeting the prespecified screening criterion for consideration in multivariable modelling (unadjusted LR p <0.20); ^a^Nominal predictors are presented as level‑versus‑reference contrast with the reference as the first coded level; ^b^Multi-categorical predictors were analysed as nominal categorical variables with the reference as the first category;* ***^c^S****cale-like ordinal predictors were treated as numeric scores, yielding a single odds ratio (OR) representing the change in odds per one‑unit increase.* | | | | |
